# Supplementary figures and images for: Towards interpretable drug interaction prediction via dual-stage attention and Bayesian calibration with active learning
Source: PeerJ Comput Sci. 2025 Apr 22;11:e2847. doi: 10.7717/peerj-cs.2847 (PMC12192666; doi:10.7717/peerj-cs.2847)

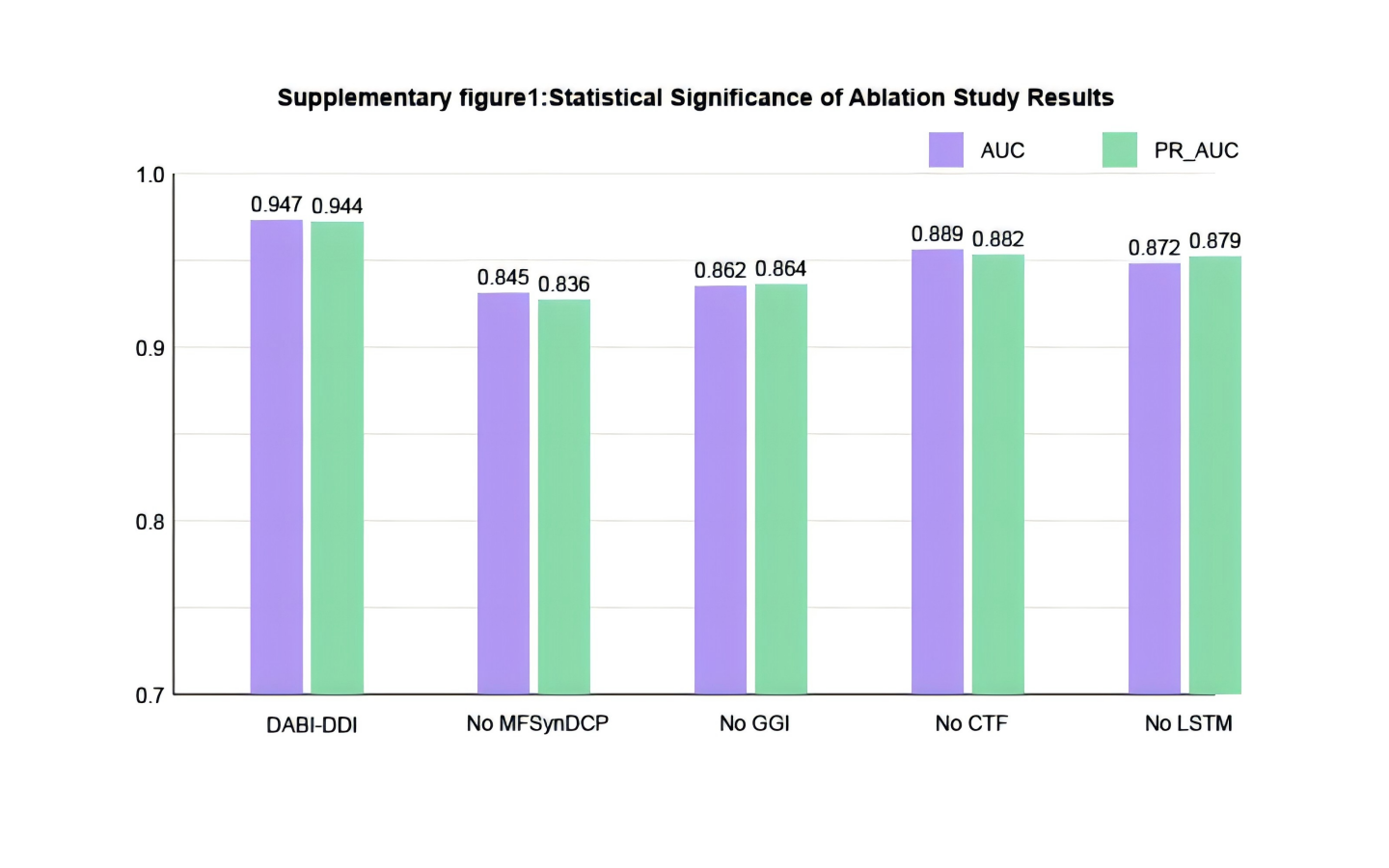

Supplement: Supplemental Information 1 — Bar plots comparing the mean AUC and PR_AUC (± standard deviation) of the full DABI-DDI model versus ablated versions across 10 independent runs. [file peerj-cs-11-2847-s001.png]

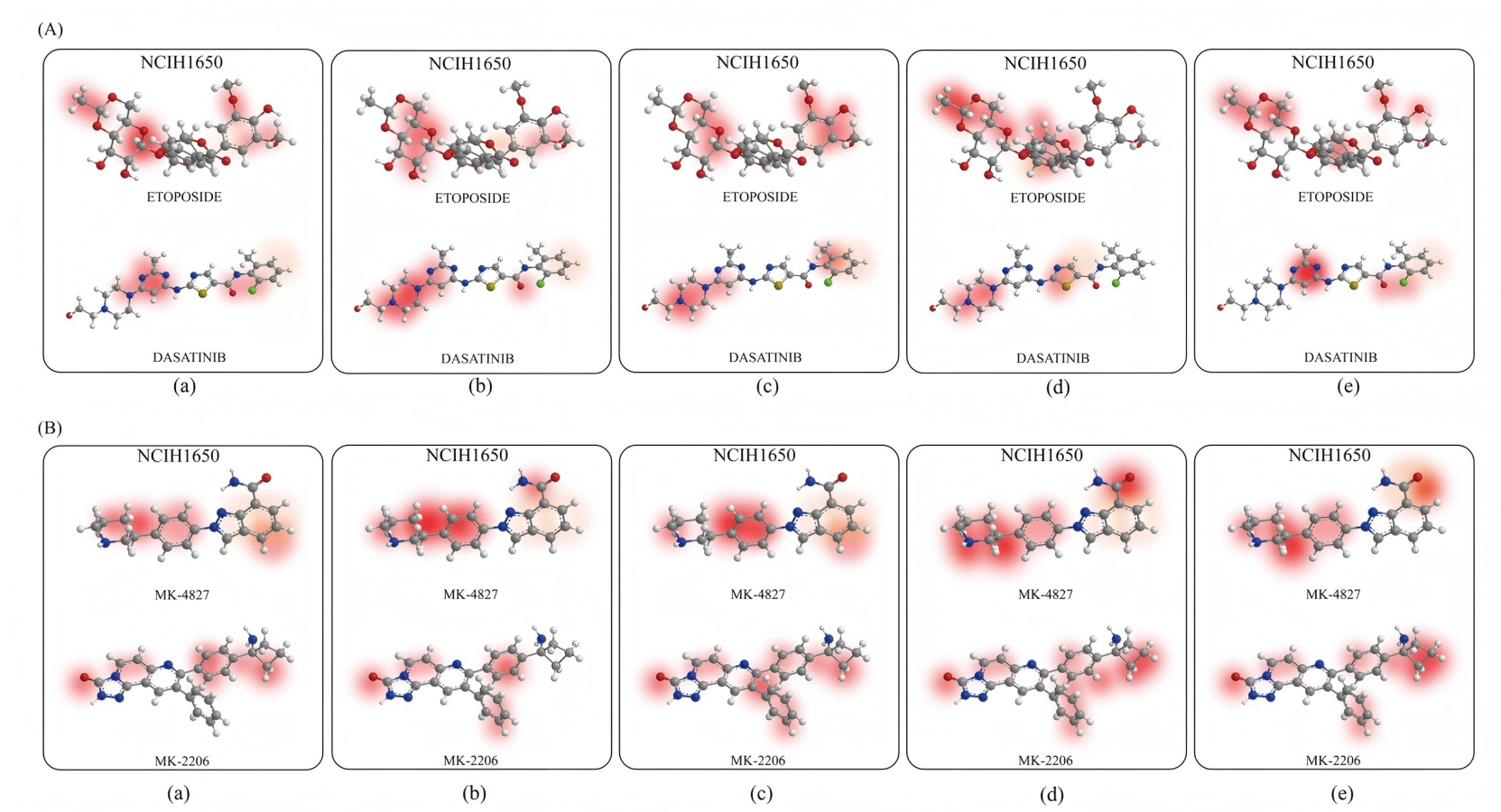

Supplement: Supplemental Information 2 [file peerj-cs-11-2847-s002.png]

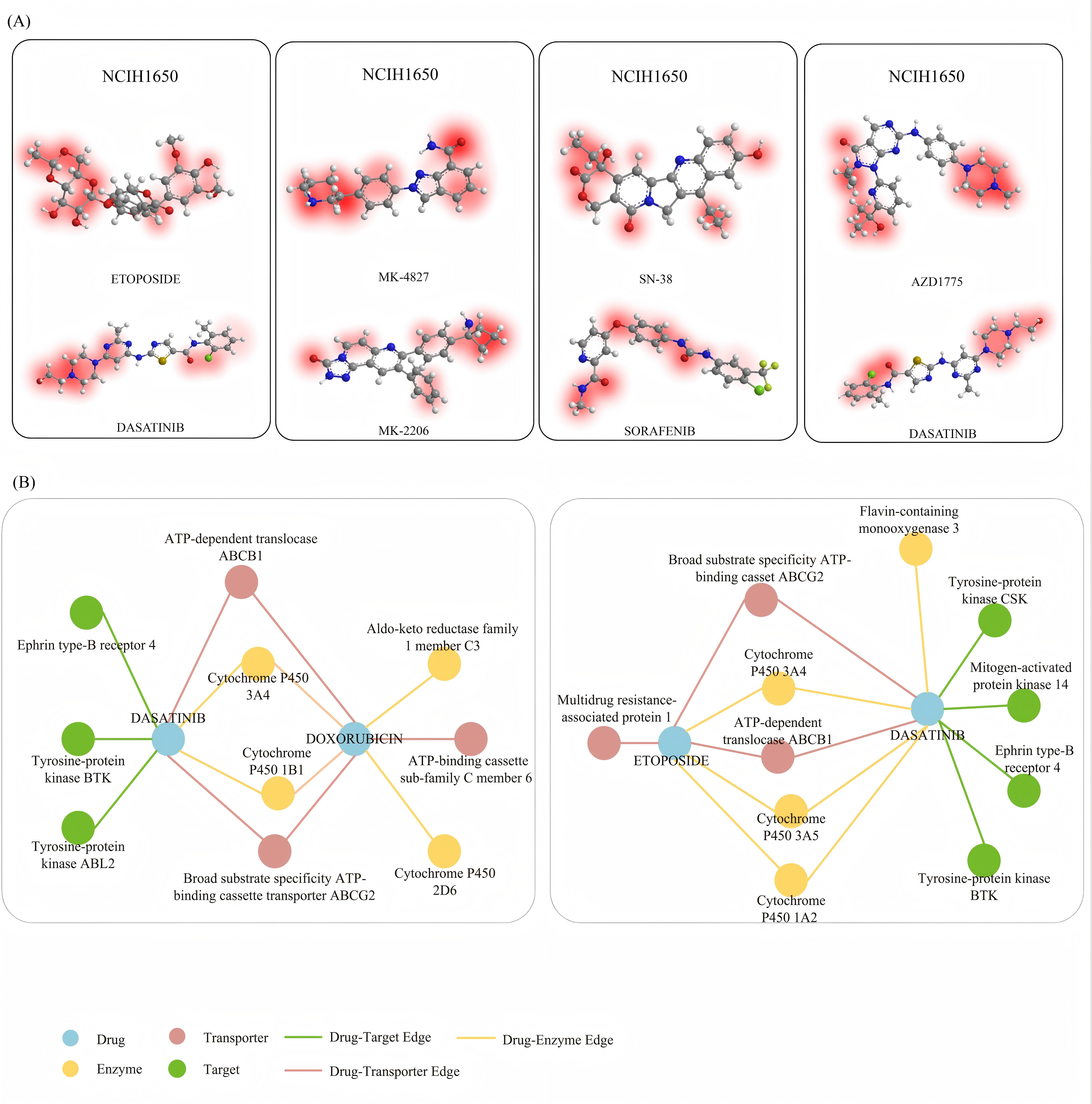

Supplement: Supplemental Information 3 — (A)The visualization results for three randomly selected drug pairs are presented. (B) Biological property attribution analysis of drug combinations. [file peerj-cs-11-2847-s003.png]
