# Supplementary material for: Towards interpretable drug interaction prediction via dual-stage attention and Bayesian calibration with active learning
Source: PeerJ Comput Sci. 2025 Apr 22;11:e2847. doi: 10.7717/peerj-cs.2847 (PMC12192666; doi:10.7717/peerj-cs.2847)
Supplement: Supplemental Information 4 [file peerj-cs-11-2847-s004.docx]

| Method | Datasets | Data Split | Preprocessing | Evaluation & Results |
| --- | --- | --- | --- | --- |
| MDF-SA-DDI (2022) | - DrugBank v5.1.8 - TWOSIDES v1.0 | - 80% train - 10% validation - 10% test | - ECFP4 molecular fingerprints - UniProt protein sequences | - 5-fold cross-validation - F1 score: 0.888 |
| MATT-DDI (2023) | - DrugBank v5.1.8 - STRING v11.5 - FAERS (2021Q1-2023Q1) | - Temporal split: - 70% pre-2022 - 30% post-2022 | - MedDRA term mapping - ≥10 co-occurrences filter | - AUPR: 0.974 - Held-out test evaluation |
| SubGE-DDI (2024) | - PubMed - DrugBank - DeepDDI benchmarks | - 90% train - 10% test | - Drug/AE entity recognition - Subgraph extraction | - 5-fold cross-validation - F1 score: 0.847 |
| MFSynDCP (2024) | - NCI-ALMANAC - CCLE | - Cell-line splits: - 80% train - 20% test | - Z-score normalized expression - RDKit drug features | - AUROC: 0.930 - Synergy prediction |
