# Supplementary material for: Towards interpretable drug interaction prediction via dual-stage attention and Bayesian calibration with active learning
Source: PeerJ Comput Sci. 2025 Apr 22;11:e2847. doi: 10.7717/peerj-cs.2847 (PMC12192666; doi:10.7717/peerj-cs.2847)
Supplement: Supplemental Information 5 [file peerj-cs-11-2847-s005.docx]

| Method | Dataset | Sample Selection | Results |
| --- | --- | --- | --- |
| Brandenburg (2023) | 1,200 surgical frames | 10% active selection | Reduced annotation effort |
| Guo (2023) | 10,000 drug pairs | Uncertainty sampling | 93% accuracy improvement |
| Raju (2023) | DFT nanocluster data | Active DFT calculation | 50-60% computation reduction |
| Liu (2023) | 3,000 TB images | Informative sampling | 83.91% accuracy |
