# Supplementary material for: Towards interpretable drug interaction prediction via dual-stage attention and Bayesian calibration with active learning
Source: PeerJ Comput Sci. 2025 Apr 22;11:e2847. doi: 10.7717/peerj-cs.2847 (PMC12192666; doi:10.7717/peerj-cs.2847)
Supplement: Supplemental Information 6 [file peerj-cs-11-2847-s006.docx]

| Method | Dataset | Approach | Performance Gain |
| --- | --- | --- | --- |
| Kontsioti (2024) | FAERS (2010-2023) | Drug-target associations | AUC +16.5% |
| Zhan (2020) | FAERS (2004-2018) | Bayesian network | 54.45% verified DDIs |
| Tada (2024) | Simulated data | Power prior method | Sensitivity +20% |
