# Supplementary material for: Towards interpretable drug interaction prediction via dual-stage attention and Bayesian calibration with active learning
Source: PeerJ Comput Sci. 2025 Apr 22;11:e2847. doi: 10.7717/peerj-cs.2847 (PMC12192666; doi:10.7717/peerj-cs.2847)
Supplement: Supplemental Information 7 [file peerj-cs-11-2847-s007.docx]

| Method | Dataset | Approach | Results |
| --- | --- | --- | --- |
| Manivannan (2023) | PDB binding data | Molecular docking | -4.23 to -4.49 kcal/mol |
| Eina (2024) | 10,000 marketing records | Feature selection | Accuracy: 75.01% → 77.88% |
