# Supplementary material for: Towards interpretable drug interaction prediction via dual-stage attention and Bayesian calibration with active learning
Source: PeerJ Comput Sci. 2025 Apr 22;11:e2847. doi: 10.7717/peerj-cs.2847 (PMC12192666; doi:10.7717/peerj-cs.2847)
Supplement: Supplemental Information 8 [file peerj-cs-11-2847-s008.docx]

| Fold | Model | PR_AUC | AUC | BACC | ACC | PREC | MSE | RMSE | F1_Score | RECALL |
| --- | --- | --- | --- | --- | --- | --- | --- | --- | --- | --- |
| 1 | DABI-DDI | 0.954 | 0.958 | 0.898 | 0.898 | 0.889 | 0.08 | 0.283 | 0.897 | 0.905 |
|  | DeepSynergy(Avg) | 0.864 | 0.882 | 0.798 | 0.801 | 0.821 | 0.166 | 0.407 | 0.777 | 0.738 |
|  | XGBoost(Avg) | 0.847 | 0.852 | 0.771 | 0.77 | 0.76 | 0.23 | 0.479 | 0.771 | 0.781 |
| 2 | DABI-DDI | 0.947 | 0.945 | 0.875 | 0.875 | 0.891 | 0.101 | 0.318 | 0.873 | 0.856 |
|  | DeepSynergy(Avg) | 0.864 | 0.882 | 0.798 | 0.801 | 0.821 | 0.166 | 0.407 | 0.777 | 0.738 |
|  | XGBoost(Avg) | 0.847 | 0.852 | 0.771 | 0.77 | 0.76 | 0.23 | 0.479 | 0.771 | 0.781 |
| 3 | DABI-DDI | 0.881 | 0.906 | 0.816 | 0.81 | 0.738 | 0.158 | 0.397 | 0.82 | 0.922 |
|  | DeepSynergy(Avg) | 0.864 | 0.882 | 0.798 | 0.801 | 0.821 | 0.166 | 0.407 | 0.777 | 0.738 |
|  | XGBoost(Avg) | 0.847 | 0.852 | 0.771 | 0.77 | 0.76 | 0.23 | 0.479 | 0.771 | 0.781 |
| 4 | DABI-DDI | 0.944 | 0.947 | 0.879 | 0.879 | 0.876 | 0.094 | 0.307 | 0.88 | 0.884 |
|  | DeepSynergy(Avg) | 0.864 | 0.882 | 0.798 | 0.801 | 0.821 | 0.166 | 0.407 | 0.777 | 0.738 |
|  | XGBoost(Avg) | 0.847 | 0.852 | 0.771 | 0.77 | 0.76 | 0.23 | 0.479 | 0.771 | 0.781 |
| 5 | DABI-DDI | 0.914 | 0.926 | 0.856 | 0.855 | 0.846 | 0.12 | 0.347 | 0.862 | 0.914 |
|  | DeepSynergy(Avg) | 0.864 | 0.882 | 0.798 | 0.801 | 0.821 | 0.166 | 0.407 | 0.777 | 0.738 |
|  | XGBoost(Avg) | 0.847 | 0.852 | 0.771 | 0.77 | 0.76 | 0.23 | 0.479 | 0.771 | 0.781 |
