# Supplementary material for: Towards interpretable drug interaction prediction via dual-stage attention and Bayesian calibration with active learning
Source: PeerJ Comput Sci. 2025 Apr 22;11:e2847. doi: 10.7717/peerj-cs.2847 (PMC12192666; doi:10.7717/peerj-cs.2847)
Supplement: Supplemental Information 9 — ¹ Ablation Study Results: Impact of Different Model Components on Performance. ²MFSynDCP - Multi-Feature Synergy Drug Combination Prediction. ³GGI - Granule-Granule Interaction. ⁴CTF - Constrained Tensor Factorization. ⁵LSTM - Long Short-Term Memory. ⁶Bold values indicate best performance. [file peerj-cs-11-2847-s009.docx]

| **Model** | **PR_AUC** | **AUC** | **BACC** | **ACC** | **PREC** | **MSE** | **RMSE** | **F1_score** | **recall** |
| --- | --- | --- | --- | --- | --- | --- | --- | --- | --- |
| **DABI-DDI** | **0.944** | **0.947** | **0.879** | **0.879** | **0.876** | **0.094** | **0.307** | **0.880** | **0.884** |
| No MFSynDCP**²** | 0.836 | 0.845 | 0.761 | 0.761 | 0.76 | 0.214 | 0.462 | 0.762 | 0.763 |
| No GGI**³** | 0.864 | 0.862 | 0.771 | 0.771 | 0.742 | 0.191 | 0.438 | 0.785 | 0.833 |
| No CTF⁴ | 0.882 | 0.889 | 0.809 | 0.809 | 0.791 | 0.173 | 0.416 | 0.815 | 0.839 |
| No LSTM⁵ | 0.879 | 0.872 | 0.793 | 0.793 | 0.797 | 0.185 | 0.43 | 0.792 | 0.788 |
| No Active Learning | 0.801 | 0.814 | 0.728 | 0.729 | 0.697 | 0.204 | 0.452 | 0.751 | 0.815 |
| No Bayesian Correction | 0.837 | 0.844 | 0.76 | 0.76 | 0.733 | 0.21 | 0.458 | 0.773 | 0.819 |
