# Supplementary material for: Towards interpretable drug interaction prediction via dual-stage attention and Bayesian calibration with active learning
Source: PeerJ Comput Sci. 2025 Apr 22;11:e2847. doi: 10.7717/peerj-cs.2847 (PMC12192666; doi:10.7717/peerj-cs.2847)
Supplement: Supplemental Information 10 — Detailed paired t-test results (t-statistics, p-values, 95% confidence intervals) for all metrics. Performance drops in ablated models are statistically significant, confirming the necessity of each component. [file peerj-cs-11-2847-s010.docx]

| Component | t-statistic | p-value | 95% CI |
| --- | --- | --- | --- |
| MFSynDCP | 12.45 | < 0.001 | [0.087, 0.117] |
| GGI | 10.83 | < 0.001 | [0.072, 0.098] |
| CTF | 8.91 | < 0.01 | [0.048, 0.068] |
| LSTM | 9.24 | < 0.01 | [0.062, 0.088] |
