# Supplementary material for: Towards interpretable drug interaction prediction via dual-stage attention and Bayesian calibration with active learning
Source: PeerJ Comput Sci. 2025 Apr 22;11:e2847. doi: 10.7717/peerj-cs.2847 (PMC12192666; doi:10.7717/peerj-cs.2847)
Supplement: Supplemental Information 11 [file peerj-cs-11-2847-s011.docx]

| Model | PR_AUC | AUC | BACC | ACC | PREC | MSE | rmse | F1_score | recall |
| --- | --- | --- | --- | --- | --- | --- | --- | --- | --- |
| DABI-DDI | 0.944 | 0.947 | 0.879 | 0.879 | 0.876 | 0.094 | 0.307 | 0.880 | 0.884 |
| No MFSynDCP+GGI | 0.658 | 0.665 | 0.604 | 0.604 | 0.612 | 0.302 | 0.549 | 0.615 | 0.618 |
| No MFSynDCP+CTF | 0.673 | 0.694 | 0.612 | 0.612 | 0.609 | 0.315 | 0.561 | 0.614 | 0.622 |
| No MFSynDCP+LSTM | 0.738 | 0.744 | 0.672 | 0.672 | 0.698 | 0.212 | 0.461 | 0.696 | 0.705 |
| No GGI+CTF | 0.785 | 0.802 | 0.718 | 0.718 | 0.706 | 0.208 | 0.456 | 0.712 | 0.721 |
| No GGI+LSTM | 0.752 | 0.764 | 0.695 | 0.695 | 0.688 | 0.224 | 0.473 | 0.705 | 0.714 |
| No CTF+LSTM | 0.781 | 0.793 | 0.719 | 0.719 | 0.709 | 0.215 | 0.464 | 0.698 | 0.715 |
