# Supplementary material for: Towards interpretable drug interaction prediction via dual-stage attention and Bayesian calibration with active learning
Source: PeerJ Comput Sci. 2025 Apr 22;11:e2847. doi: 10.7717/peerj-cs.2847 (PMC12192666; doi:10.7717/peerj-cs.2847)
Supplement: Supplemental Information 12 [file peerj-cs-11-2847-s012.docx]

| Analysis Aspect | Details |
| --- | --- |
| **Case Study 1: Drug Combination** | Sotalol + Moxonidine |
| Drug Classes | • Sotalol: β-blocker & K+ channel inhibitor • Moxonidine: Selective I1-imidazoline receptor agonist (central α2-adrenergic agonist) |
| Mechanisms & Interaction | • Sotalol reduces heart rate and blood pressure via β-blockade and potassium channel inhibition • Moxonidine lowers blood pressure by reducing sympathetic outflow in CNS • **Synergistic Risk**: Combined effects on sympathetic activity and vasodilation amplify hypotension |
| Clinical Evidence | Both drugs are known to cause hypotension individually. Their co-administration is contraindicated in some guidelines due to additive hypotensive effects |
| Model Alignment | • High Bayesian posterior probability (score = 0.72) • Active learning flagged as uncertain during training • Enzyme-transporter pathway analysis linked to CYP3A4-mediated metabolism and ABCB1 transporter competition |
| **Case Study 2: Drug Combination** | Amitriptyline HCl + Imipramine HCl |
| Drug Classes | Both Amitriptyline and Imipramine are Tricyclic antidepressants (TCAs) |
| Mechanisms & Interaction | • Both TCAs inhibit norepinephrine reuptake and block α1-adrenergic receptors, leading to vasodilation • **Synergistic Risk**: Combined α1 blockade exacerbates orthostatic hypotension |
| Clinical Evidence | • TCAs are rarely co-prescribed due to well-documented additive cardiovascular risks • Case reports highlight severe hypotension in polypharmacy scenarios |
| Model Alignment | • Dual-stage attention mechanism identified shared aromatic and heterocyclic substructures • Bayesian calibration reduced false positives by incorporating prior knowledge of TCA-related adverse events |
| **Case Study 3: Drug Combination** | Haloperidol Decanoate + Methyldopa |
| Drug Classes | • Haloperidol: Typical antipsychotic (dopamine D2 receptor antagonist) • Methyldopa: Centrally acting α2-adrenergic agonist |
| Mechanisms & Interaction | • Methyldopa lowers blood pressure via central α2 agonism and reduced peripheral resistance • Haloperidol causes hypotension through α1-adrenergic blockade and QT prolongation • **Synergistic Risk**: Combined α-adrenergic effects and CNS depression amplify hypotension |
| Clinical Evidence | Retrospective studies report hypotension in patients co-prescribed antipsychotics and antihypertensives |
| Model Alignment | • Network pharmacology analysis revealed interactions via CYP2D6 and ABCG2 transporters • LSTM module captured temporal patterns in FAERS reports where hypotension emerged weeks after combination therapy initiation |
