# Supplementary material for: Towards interpretable drug interaction prediction via dual-stage attention and Bayesian calibration with active learning
Source: PeerJ Comput Sci. 2025 Apr 22;11:e2847. doi: 10.7717/peerj-cs.2847 (PMC12192666; doi:10.7717/peerj-cs.2847)
Supplement: Supplemental Information 14 [file peerj-cs-11-2847-s014.docx]

# Documentation

## Description of the files

This project consists of two main subfolders: **Mymodel** and **Bayes**, which demonstrate the construction and application of deep learning models and Bayesian probabilistic models, respectively.

In the **Mymodel** folder, we organize the model files (in the **models** folder), data files (in the **data** folder), dataset creation scripts, and model training scripts separately for better structure and accessibility. The **creat_data.py** file is used to directly create the dataset required for model execution. The **mymodel_train.py** file contains detailed training procedures for all models, while the **BAN.py** file is designed to visualize the biological attribute network.

## Description of the data

This project primarily utilizes the **FAERS**, **DrugBank**, **Open Targets**, and **STRING** datasets. For the **FAERS** dataset, we apply the **AEOLUS** standardization process to clean and harmonize the data. Reports lacking information on drugs or adverse events (AEs) and ambiguous data that cannot be standardized are excluded. Drug names are mapped to **RxNorm ingredient-level terms**, and AE descriptions are standardized to **MedDRA Preferred Terms (PTs)** to ensure data consistency and comparability. Regarding the **DrugBank** dataset, we download version **5.1.9** in XML format and extract chemical properties (such as molecular weight, hydrogen bond count, and atomic composition) and pharmacokinetic parameters (including bioavailability, half-life, and protein binding rate). Drug names and their **SMILES** representations are then converted into a CSV file for further analysis. For the **Open Targets** dataset, we extract drug-target and target-AE associations to assess potential safety risks. Drug names are mapped to **RxNorm ingredient-level terms**, while targets are identified using **Ensembl stable IDs** and **UniProtKB accession numbers** to ensure standardized and traceable data. From the **STRING** dataset, we select protein-protein interactions (PPIs) specific to humans with a confidence score above a defined threshold, ensuring data reliability. These high-confidence PPIs provide valuable insights into the molecular mechanisms of drug interactions, enhancing the credibility of our research findings. In this study, we directly utilize a dataset curated by **E. Kontsioti et al. [1]**, which includes the following fields: **drug1**, **drug2**, **event**, **n_111**, **n_101**, **n_011**, **n_001**, **d1_d2_counter**, **d1_not_d2_counter**, **not_d1_d2_counter**, and **not_d1_not_d2_counter**. The detailed definitions and interpretations of these fields are provided below.：

- **drug1 & drug2**: The names of the two interacting drugs.
- **event**: The name of the adverse event observed.
- **n_111**: The total sample size of individuals concurrently using both drugs.
- **n_101**: The total sample size of individuals using **drug1** but not **drug2**.
- **n_011**: The total sample size of individuals not using **drug1** but using **drug2**.
- **n_001**: The total sample size of individuals not using either **drug1** or **drug2**.
- **d1_d2_counter**: The number of adverse events observed among individuals concurrently using both drugs.
- **d1_not_d2_counter**: The number of adverse events observed among individuals using **drug1** but not **drug2**.
- **not_d1_d2_counter**: The number of adverse events observed among individuals not using **drug1** but using **drug2**.
- **not_d1_not_d2_counter**: The number of adverse events observed among individuals not using either **drug1** or **drug2**.

To train our deep learning model for the DDI task, we used the large-scale tumor screening drug combination dataset published by **O'Neil et al. [2]** in 2016 as the foundational dataset. This dataset primarily includes the variables **cell_line**, **drugA_name**, **drugB_name**, and a variable **X** that describes the interaction between the drugs. The definitions of these fields are as follows:

- **cell_line**: The specific cancer cell line on which the drug combination experiments were conducted.
- **drugA_name**: The name of the first drug in the combination.
- **drugB_name**: The name of the second drug in the combination.
- **X**: The confidence score representing the synergistic effect between the two drugs. A higher value of **X** indicates a stronger synergistic interaction between the drugs.

## Description of the Code Execution

All experimental codes for the **Bayes model** can be executed directly by running the **bayes_train.py** file located in the **Bayes** folder. By executing this file, the posterior probabilities of adverse events derived from the FAERS dataset using the Bayesian model will be obtained. Additionally, a comparison plot of AUC curves between the Bayesian model and the signal detection algorithm is generated. The output also includes the probabilities of adverse events associated with various drug combinations and their corresponding confidence levels. This enables the identification of drug combinations with a high confidence of causing adverse events through the Bayesian approach.

For all experiments related to the **Mymodel** model, the dataset can first be created using the **create_data.py** file in the **Mymodel** folder. The model training and validation process can then be performed by running the **mymodel_train.py** file. All experimental results are saved in the **result** folder, including the comparative performance of the proposed model against other models, ablation study results, and ROC curve comparison plots. The biological attribute network can be visualized by directly executing the **BAN.py** file.

All data processing and model training were conducted on an **Ubuntu 20.04 LTS** system, and the codebase was developed using the **Torch** framework. The required Python libraries for running the experiments are listed in the **requirement.txt** file.

[1]Kontsioti E, Maskell S, Anderson I, et al. Identifying Drug–Drug Interactions in Spontaneous Reports Utilizing Signal Detection and Biological Plausibility Aspects[J]. Clinical Pharmacology & Therapeutics, 2024, 116(1): 165-176.

[2]O’Neil J, Benita Y, Feldman I, Chenard M, Roberts B, Liu Y, et al. An unbiased oncology compound screen to identify novel combination strategies. Mol Cancer Ther. 2016;15:1155–62. https:// doi. org/ 10. 1158/ 1535- 7163. MCT- 15- 0843.
